# How Numerical Unbalance Constraints Physical and Tactical Individual Demands of Ball Possession Small-Sided Soccer Games

**DOI:** 10.3389/fpsyg.2020.01464

**Published:** 2020-07-14

**Authors:** Nuno André Nunes, Bruno Gonçalves, Diogo Coutinho, Bruno Travassos

**Affiliations:** ^1^Department of Sports Sciences, University of Beira Interior, Covilhã, Portugal; ^2^Research Centre in Sports Sciences, Health, Sciences and Human Development, CIDESD, CreativeLab Research Community, Vila Real, Portugal; ^3^Comprehensive Health Research Centre (CHRC), Universidade de Évora, Évora, Portugal; ^4^Departamento de Desporto e Saúde, Escola de Ciências e Tecnologia, Universidade de Évora, Évora, Portugal; ^5^Portugal Football School, Portuguese Football Federation, Oeiras, Portugal

**Keywords:** constraint-led approach, design of practice tasks, GPS monitoring, team sports, coaching development

## Abstract

This study aimed to explore the effects of playing different unbalanced ball possession small-sided games on external workload (distance covered while walking, running, and sprinting, and max speed), tactical individual actions (number of passes with dominant and non-dominant foot), and internal load (rating of perceived exertion, RPE) in under-23 soccer players. Participants played 4v2, 4v3, 4v4, 4v5, and 4v6 small-sided games (SSGs) on a 30 × 25 m playing area. Data were analyzed under an opponent-based perspective, by fixing one team (4vX), and by cooperation-based perspective according to teammates (4v2+X). Global Position System (GPS) monitors were used to collect and compute external workloads and individual tactical actions, and Borg Scale CR10 was used to evaluate RPE. High-Superiority (4v2), Superiority (4v3), and Very Low-Cooperation (4v2+0) formats allow players in balanced teams to cover more distance while walking; on the other side, Inferiority (4v5), High-Inferiority (4v6), and Very High-Cooperation (4v2+4) allow players to sprint more and practice more tactical individual actions as a resultant emergent behavior; all players in SSG conditions with a lower number of conditions, perceived the exercise as more intense, especially in situations with less than two players. Overall, playing in high inferiority situations (4v2 and 4v6) may be used to increase physical demand for the outnumbered team, while coaches may use low superiority situations to adjust the task complexity when developing the players’ tactical individual actions.

## Introduction

Performance in soccer is related to a player’s ability to interact with the environmental information (Travassos et al., 2012), where each player with or without the ball can continuously reach new solutions to destabilize the relation with opponents inside a collective idea of play (Davids et al., 2005). In order to develop the performance of players, coaches are challenged to provide functional and adjusted learning experiences and to provide opportunities for potential performance solutions, to be generated by themselves (Chow et al., 2016; Woods et al., 2020).

The constraints-led approach (CLA) highlights the major role of constraint manipulations in shaping player and team performance, from training tasks to competitive environments (Chow et al., 2016). That is, the manipulation of task constraints should highlight perceptual-action relations between players and the game environment, which allows for the development of their attunement (i.e., capability to continuously pick up the information that supports players’ actions) and calibration (i.e., capability to functionally adjust each action to spatial-temporal relations with teammates and opponents) (Araújo et al., 2009). Accordingly, small-sided games (SSGs) are often used to simulate full game or game phases, maintaining their unstable, dynamic and unpredictable nature, while coupling players actions to the available information (Davids et al., 2013; Travassos et al., 2014a; Coutinho et al., 2018a, b, 2019).

Furthermore, the manipulation of SSGs seems to promote changes not only in tactical but also in the external and internal load of players (Hill-Haas et al., 2010). For instance, through the monitoring of internal load using the Borg Rating of Perceived Exertion scale (RPE) and the external load using Global Position System (GPS) in complete football training sessions, Casamichana et al. (2013) found significant correlations between RPE and most of the external indicators studied. In addition, players are likely to modify their performance as result of the perceived effort. As such, manipulations of SSGs promote changes in players’ perception of efforts, with consequences in pacing and in the individual tactical behavior of players (Marcora, 2008, 2009).

Despite increasing research on SSGs in the last years, few studies have focused the attention on numerical unbalance between teams, even though soccer is often played in unbalanced numbers (Torres-Ronda et al., 2015; Rabano-Munoz et al., 2019). Even as a strategic component, there are game phases in which the numerical superiority is sought by the teams to create goal scoring opportunities and to prevent opponents from scoring (Evangelos et al., 2012). Moreover, numerical unbalance can be used as a reliable constraint to improve the learning/training process using SSGs due to the required flexibility on the tactical solutions, as it seems to emphasize local information that players should attend to, to unfold goal-directed behaviors (Ric et al., 2016).

Since soccer is often played with a numerical unbalance, coaches should seek to encourage players to explore interpersonal interactions between teammates and opponents during the training situations of numerical inferiority and superiority (Torres-Ronda et al., 2015). Previous investigations regarding physical and physiological demands of SSGs on this topic, reported that although the team with the numerical inferiority perceives the most intense exercise compared with the team with superiority, no significant differences were observed in the external load and physiological responses (Hill-Haas et al., 2010). In contrast, more recent studies on opposition- and cooperation-based perspectives, reported that playing with minimal inferiority (4v3 and 4v5) was more physiologically demanding than with high inferiority (4v7) (Torres-Ronda et al., 2015). Furthermore, when playing with high inferiority (4v7), players tend to promote team-related auto-organization, by staying compact and working as a unit, as in a performance context (Gonçalves et al., 2016).

The use of a numerical unbalance highlights specific spaces and actions according to the advantage/disadvantage of play and encourages the emergence of new game patterns and individual tactical actions (Sarmento et al., 2018). For example, Vilar et al. (2014b) reported that while the practice with one less defender (5v4) does not impact the defensive team’s ability to intercept passes or team’s shots, the practice with two less defenders (5v3) induced significant changes in the behavior of the attackers, leading them to create more opportunities for shooting and scoring, as well as passing among the players.

Overall, research in SSGs accounting for unbalanced situations has shown distinct physical, technical, and tactical behaviors according to numerical inequality (Travassos et al., 2014b; Vilar et al., 2014b; Torres-Ronda et al., 2015; Gonçalves et al., 2016). However, most of these studies analyzed the player’s performance during game-based situations using regular goals. Accordingly, during the last few years there has been an increase in the popularity of these game-based drills, on the basis that it allows improving passing actions and space occupation by the attacking team, or space equilibrium and compactness by the defensive team. So, further research is required to improve the understanding of the manipulation of numerical unbalance, in a systematic way, on responses of soccer players.

The aim of this study was then to evaluate the effects of the manipulation of an unbalanced number of players (4v2, 4v3, 4v4, 4v5, and 4v6), through opposition- and cooperation-based perspectives, on players’ physical and individual tactical performance. We expected higher values of internal and external workload for players in situations of numerical inferiority in relation to the opponent team (Kalapotharakos et al., 2011). Higher values of internal and external loads in relation to cooperation between players is expected, the higher the number of players involved in the task is (Torres-Ronda et al., 2015). In terms of tactical individual actions, we expect that teams in numerical inferiority perform a lower number of passes as they may have more difficulty in recovering ball possession and also when attacking, due to the difficulty to create available passing lines.

## Materials and Methods

### Participants

Twenty university-level soccer players, playing at a semi-professional level participated in this study (age 22.3 ± 2.0 years, body mass: 71.4 ± 7.0 kg, height: 177.1 ± 6.8 cm, years of experience: 12.1 ± 3.7 years, 13 players with a dominant right foot and seven with a dominant left foot). All participants were part of the same team and experienced three weekly 90-min training sessions (which included technical drills, fitness exercises, and game-based tasks) plus one game on weekends at a regional playing standard in a regular turf football field. Players have around 40 weeks of training per sporting season and no goalkeepers were included for data collection. The experimental protocol and investigation were approved by the local Institutional Research Ethics Committee and was performed in accordance with the ethical standards of the Helsinki Declaration. Written, informed consent was obtained for all participants, the coach, and the club before data collection commenced.

### Procedures

**FIGURE 1:**
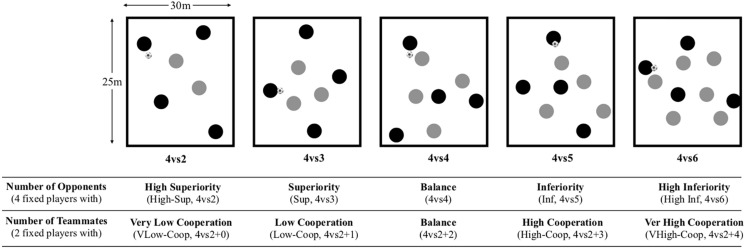
Small-sided games study design.

Participants performed in a 4-series of 4vsX (2, 3, 4, 5, and 6) SSGs (Abrantes et al., 2012; Aguiar et al., 2013) for ball possession on a 30 × 25 m playing area in an artificial turf pitch (Owen et al., 2004; Williams and Owen, 2007). Due to the purpose of the exercise (maintaining and recovering ball possession), no goalkeeper or any type of goal or target was used. Data was collected in one single session, during the competitive period. From the opposition-based perspective, variables were analyzed by fixing the same four players and comparing them against two (High Superiority; High-Sup, 4v2), three (Superiority; Sup, 4v3), four (Balance, 4v4), five (Inferiority; Inf, 4v5), and six (High Inferiority; High-Inf, 4v6) players; in the cooperation-based perspective, variables were analyzed by comparing performances from the same two players when counting with none (Very Low; VLow-Coop, 4v2+0), one (Low; Low-Coop, 4v2+1), two (Balance, 4v2+2), three (High; High-Coop, 4v2+3), and four (Very High: VHigh-Coop, 4v2+4) teammates (Figure 1; Torres-Ronda et al., 2015; Gonçalves et al., 2016). Each SSG format (4v2, 4v3, 4v4, 4v5, and 4v6) was performed for four sets of 4 min each (in a total of 16 min of intermittent exercise for each SSG format; 4 × 4 min + 4 min recovery time) (Halouani et al., 2014). These procedures allowed us to collect a total of 20 SSGs, each player taking part in around eight of them. Between bouts, players were asked to perform some lower limb stretching exercises according to their individual preference. The different SSG formats followed a random order. Before the beginning of each session, players performed a general warm-up that included running at various intensities, joint mobilization, and stretching of 20-min duration. The coach did not intervene with any corrective feedback during the SSG. If the ball went out of play, other strategically placed balls allowed an immediate restart from a pass.

### Data Collection

Data on the external workload variables were collected through a GPS included in the ZEPP Play Soccer system (ZEPP Labs, San Jose, United States), which uses two Micro Electromechanical Systems (MEMS) sensors and Bluetooth 4.0 Low Energy (LE) connectivity. Each player had a microchip (each with two internal sensors: 3-Axis Accelerometer + 3-Axis Gyroscope) attached to each of their gastrocnemius to record displacement data (Aroganam et al., 2019; Sasaki et al., 2019). Later, Zepp’s computer software (version 1.6.0) was used to compute the values of total distance covered (m), distance differentiated by walking (≤9 km/h), running (9–18 km/h), and sprinting (>18 km/h), number of sprints (n), maximum sprint speed (km/h), number of passes (ball contacts) with the dominant and non-dominant foot (n), and maximum pass speed (km/h) (Owen et al., 2014). The internal load perceptions were measured using a Borg Scale CR10 to evaluate RPE. It was presented to participants after the end of each SSG bout to ensure that the perceived effort was referring to that specific game (Coutts et al., 2009). For the tactical individual actions of passing, it was counted when the force applied to the ball allowed it to travel a distance of at least 5 m, using ZEPP Play Soccer system.

### Statistical Analysis

The statistical analysis was completed using the Statistical Package for Social Sciences software V24.0 (IBM SPSS Statistics for Windows, Armonk, NY, United States: IBM Corp.). A descriptive analysis was graphically performed using box plots and the means with SD were reported in text. The pairwise comparisons were carried out according to two scenarios: (i) opposition-based perspective, where the effects of changing the number of opponents was analyzed; and (ii) cooperation-based perspective, where the effects of changing the number of teammates was analyzed. All differences were assessed *via* standardized differences with pooled variance, derived from the mean and standard deviation of each variable, with 95% confidence intervals (95% CI). The magnitude thresholds for effect size of mean differences (Cohen’s d) were 0.20, 0.60, 1.2, 2.0, and 4.0 for small, moderate, large, very large, and extremely large, respectively (Cumming, 2012).

## Results

### Opposition-Based Perspective

**FIGURE 2:**
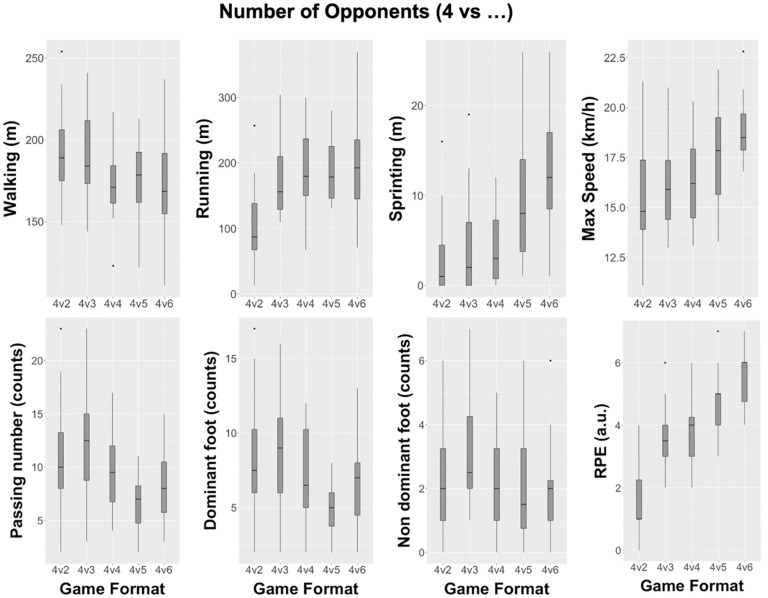
Descriptive values (box plots) when considering the variation of the number of opponents.

Figure 2 depicts the descriptive values (box plots) of external load, perception of internal load, and tactical individual actions when considering the variation of the number of opponents. Complementary Table 1 presents the descriptive values (mean ± SD), mean difference with 95% confidence intervals, and the Cohen’s d with 95% confidence intervals of each variable. Differences were found between High-Sup and Balance formats (4v2 vs 4v4), with a moderate effect size on walking [−0.9 (−1.7 to −0.1), Cohen’s d with 95% confidence intervals] and large effect size in both running [1.4 (0.7 to 2.1)] and RPE [1.9 (1 to 2.8)]; and between Sup and Balance formats (4v3 vs 4v4), with moderate effects on walking [−0.7 (−1.4 to 0.1)]. The higher number of differences were observed between Balance and Inf formats (4v4 vs 4v5), with moderate higher distances while sprinting [0.9 (0.1 to 1.7)], moderately fewer passes [−1 (−1.8 to −0.1)], moderately fewer passes with the dominant foot [−1.1 (−1.9 to −0.2)], and moderately higher RPE [0.8 (0.2 to 1.4)] in unbalanced format (4v5); and between Balance and High-Inf formats (4v4 vs 4v6) with large higher distances while sprinting [1.4 (0.6 to 2.1)], large higher max speed [1.4 (0.5 to 2.2)] and large RPE [1.4 (0.7 to 2.2)] also in unbalanced conditions (4v6) (see Table 1).

**TABLE 1:** Descriptive values (mean ± SD), mean difference with 95% confidence intervals and Cohen’s d with 95% confidence intervals for an opposition-based perspective.

Variables	Game format					Mean difference with 95% of confidence intervals Cohen’s d with 95% of confidence intervals			
	4v2	4v3	4v4	4v5	4v6	4v2 vs 4v4	4v3 vs 4v4	4v4 vs 4v5	4v4 vs 4v6
	High-Sup	Sup	Balance	Inf	High-Inf	High-Sup vs Balance	Sup vs Balance	Balance vs Inf	Balance vs High-Inf
**External workload**									
Walking (m)	193.9 ± 26	189.9 ± 27.9	173 ± 22.1	176.8 ± 23.6	173.1 ± 33	**−20.9 (−40.2 to −1.7)**	**−16.9 (−35.5 to 1.6)**	3.8 (−14.1 to 21.7)	0.1 (−17.1 to 17.2)
						**−0.9 (−1.7 to −0.1)**	**−0.7 (−1.4 to 0.1)**	0.2 (−0.6 to 0.9)	0 (−0.6 to 0.6)
Running (m)	102.3 ± 64.3	173 ± 56.7	191.7 ± 61.3	187.7 ± 47.3	199.2 ± 81.6	**89.4 (54.3 to 124.5)**	18.7 (−23.7 to 61.1)	−4 (−35.9 to 27.9)	7.5 (−43.6 to 58.6)
						**1.4 (0.7 to 2.1)**	0.3 (−0.4 to 1)	−0.1 (−0.6 to 0.5)	0.1 (−0.6 to 0.8)
Sprinting (m)	3.3 ± 4.6	4.7 ± 5.8	4.2 ± 4.2	9.9 ± 8	11.8 ± 6.6	0.9 (−1.4 to 3.3)	−0.5 (−3.9 to 2.9)	**5.8 (0.9 to 10.6)**	**7.6 (4.4 to 10.8)**
						0.2 (−0.3 to 0.7)	−0.1 (−0.7 to 0.5)	**0.9 (0.1 to 1.7)**	**1.4 (0.6 to 2.1)**
Max speed (km/h)	15.6 ± 3	16.2 ± 2.4	16.3 ± 2.2	17.5 ± 2.6	18.9 ± 1.5	0.8 (−0.9 to 2.4)	0.1 (−1.4 to 1.6)	1.2 (−0.8 to 3.1)	**2.6 (1.2 to 4)**
						0.3 (−0.3 to 0.9)	0.1 (−0.6 to 0.7)	0.5 (−0.3 to 1.2)	**1.4 (0.5 to 2.2)**
**Tactical individual actions**									
Passing number (count)	11.1 ± 5.4	12 ± 5.2	9.8 ± 3.8	6.4 ± 2.7	8.5 ± 3.8	−1.3 (−4.7 to 2.1)	−2.3 (−6.4 to 1.9)	**−3.3 (−6 to −0.6)**	−1.3 (−3.8 to 1.3)
						−0.3 (−1 to 0.4)	−0.5 (−1.3 to 0.4)	**−1 (−1.8 to −0.1)**	−0.3 (−1 to 0.3)
Dominant foot (counts)	8.6 ± 4.2	8.8 ± 3.9	7.4 ± 3.2	4.7 ± 1.7	6.5 ± 3.2	−1.2 (−3.8 to 1.5)	−1.4 (−4.4 to 1.7)	**−2.8 (−4.9 to −0.6)**	−0.9 (−3.2 to 1.3)
						−0.3 (−1 to 0.3)	−0.4 (−1.2 to 0.4)	**−1.1 (−1.9 to −0.2)**	−0.3 (−0.9 to 0.4)
Non-dominant foot (counts)	2.4 ± 2	3.2 ± 1.8	2.3 ± 1.5	2 ± 1.9	2 ± 1.5	−0.1 (−1.7 to 1.4)	−0.9 (−2.4 to 0.7)	−0.3 (−1.7 to 1.1)	−0.3 (−1.2 to 0.5)
						−0.1 (−0.9 to 0.7)	−0.5 (−1.4 to 0.3)	−0.2 (−0.9 to 0.6)	−0.2 (−0.7 to 0.3)
**Internal load of perceptions**									
RPE (a.u.)	1.7 ± 1.1	3.5 ± 1.1	3.9 ± 1.2	4.8 ± 0.9	5.5 ± 1	**2.2 (1.5 to 2.9)**	0.4 (−0.3 to 1.1)	**0.9 (0.2 to 1.5)**	**1.6 (1 to 2.3)**
						**1.9 (1 to 2.8)**	0.3 (−0.3 to 0.9)	**0.8 (0.2 to 1.4)**	**1.4 (0.7 to 2.2)**

*High-Sup, High Superiority; Sup, Superiority; Inf, Inferiority; High-Inf, High Inferiority. The bold on the tables are referred to the values with effects size on the different variables and the ones consider for discussion.*

### Cooperation-Based Perspective

**FIGURE 3:**
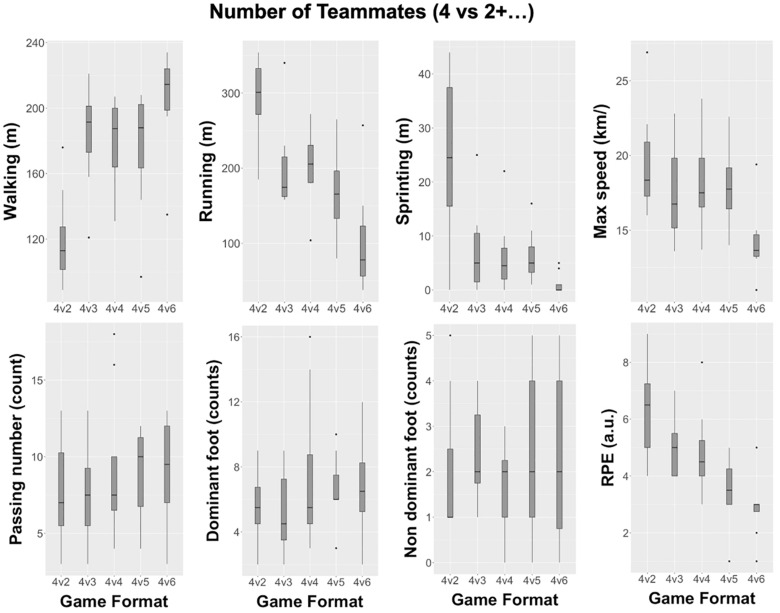
Descriptive values (box plots) when considering the variation of the number of cooperation.

Figure 3 and Table 2 depict the descriptive and inferential results when considering the variation of the number of players in cooperation. Differences were found between VLow-Coop and Balance formats (4v2+0 vs 4v4), with higher large distances on walking [2.2 (0.7 to 3.6), Cohen’s d with 95% confidence intervals], lower large effect on running [−1.6 (−2.8 to −0.5)] and sprinting [−1.4 (−2.5 to −0.2)], on unbalanced formats (4v2+0); and lower moderate effect size on RPE [−0.9 (−2 to 0.2)], with participants perceiving the exercise as more intense also in unbalanced formats (4v2+0). Participants moderately covered more distance while running [−0.7 (−2.5 to 0.1)] and also as perceived the exercise as more intense [−1 (−2.3 to 0.5)] in balanced (4v4) formats compared to High-Coop (4v2+3). The higher number of effects were found when comparing Balance (4v4) to VHigh-Coop (4v2+6) formats, with moderate effect sizes on walking [0.9 (0 to 1.8)] and sprinting [−1 (−1.9 to −0.1)], and large effect sizes on running [−1.6 (−2.7 to −0.5)], max speed [−1.4 (−2.6 to −0.2)], and RPE [−1.5 (−2.6 to −0.3)] (see Table 2).

**TABLE 2:** Descriptive values (mean ± SD), mean difference with 95% confidence intervals and Cohen’s d with 95% confidence intervals for a cooperation-based perspective.

Variables	Game format					Mean difference with 95% of confidence intervals Cohen’s d with 95% of confidence intervals			
		
	4v2 (+0)	4v3 (2+1)	4v4 (2+02)	4v5 (2+3)	4v6 (2+4)	4v2+0 vs 4v4	4v2+1 vs 4v4	4v4 vs 4v2+3	4v4 vs 4v2+4
	VLow-Coop	Low-Coop	Balance	High-Coop	VHigh-Coop	VLow-Coop vs Balance	Low-Coop vs Balance	Balance vs High-Coop	Balance vs VHigh-Coop
**External Workload**									
Walking (m)	120.1 ± 29.1	183 ± 31.1	180.4 ± 25.9	175.3 ± 38.1	205.9 ± 31.9	**60.3 (30 to 90.5)**	−2.6 (−25.6 to 20.4)	−5.1 (−46.2 to 35.9)	**25.5 (−1.7 to 52.7)**
						**2.2 (0.7 to 3.6)**	−0.1 (−0.8 to 0.6)	−0.2 (−1.2 to 0.9)	**0.9 (0 to 1.8)**
Running (m)	293.3 ± 57.1	201.3 ± 61.8	203.5 ± 52.4	164.1 ± 60.4	102.9 ± 72.2	**−89.8 (−142.5 to −37)**	2.3 (−36.1 to 40.6)	**−39.4 (−89.2 to 10.4)**	**−100.6 (−157.4 to −43.8)**
						**−1.6 (−2.8 to −0.5)**	0 (−0.5 to 0.6)	**−0.7 (−1.5 to 0.1)**	**−1.6 (−2.7 to −0.5)**
Sprinting (m)	24.1 ± 16.7	7.4 ± 8.3	6.5 ± 7	6.3 ± 5.1	1.1 ± 2.1	**−17.6 (−31.1 to −4.2)**	−0.9 (−9.5 to 7.8)	−0.3 (−7.2 to 6.7)	**−5.4 (−9.8 to −1)**
						**−1.4 (−2.5 to −0.2)**	−0.1 (−1 to 0.8)	0 (−1 to 0.9)	**−1 (−1.9 to −0.1)**
Max speed (km/h)	19.5 ± 3.6	17.5 ± 3.1	18.1 ± 3.1	17.8 ± 2.7	14.2 ± 2.4	−1.4 (−5.6 to 2.8)	0.7 (−1.9 to 3.2)	−0.3 (−4.1 to 3.4)	**−3.9 (−7.2 to −0.6)**
						−0.4 (−1.5 to 0.7)	0.2 (−0.5 to 0.9)	−0.1 (−1.2 to 1)	**−1.4 (−2.6 to −0.2)**
**Tactical individual actions**									
Passing number (count)	7.6 ± 3.5	7.5 ± 3.3	9.1 ± 5.1	9 ± 3	8.9 ± 3.8	1.5 (−4.3 to 7.3)	1.6 (−3.3 to 6.5)	−0.1 (−5.5 to 5.2)	−0.3 (−5.3 to 4.8)
						0.3 (−0.8 to 1.5)	0.4 (−0.6 to 1.3)	0 (−1.1 to 1)	−0.1 (−1 to 0.9)
Dominant foot (counts)	5.6 ± 2.5	5.1 ± 2.6	7.4 ± 4.9	6.6 ± 2.1	6.6 ± 3.2	1.8 (−3.1 to 6.6)	2.3 (−3 to 7.5)	−0.8 (−5 to 3.5)	−0.8 (−5.3 to 3.8)
						0.4 (−0.6 to 1.5)	0.6 (−0.6 to 1.7)	−0.2 (−1.1 to 0.7)	−0.2 (−1.1 to 0.7)
Non-dominant foot (counts)	2 ± 1.6	2.4 ± 1.2	1.8 ± 1	2.4 ± 1.8	2.3 ± 1.9	−0.3 (−1.7 to 1.2)	−0.6 (−1.7 to 0.5)	0.6 (−1.5 to 2.8)	0.5 (−1.4 to 2.4)
						−0.2 (−1.1 to 0.7)	−0.6 (−1.4 to 0.3)	0.4 (−0.8 to 1.6)	0.3 (−0.8 to 1.4)
**Internal load of perceptions**									
RPE (a.u.)	6.4 ± 1.7	5.1 ± 1.2	4.9 ± 1.6	3.5 ± 1.3	2.9 ± 1.1	**−1.5 (−3.3 to 0.3)**	−0.3 (−1.8 to 1.3)	**−1.4 (−3.7 to 0.9)**	**−2 (−3.5 to −0.5)**
						**−0.9 (−2 to 0.2)**	−0.2 (−1.1 to 0.8)	**−1 (−2.3 to 0.5)**	**−1.5 (−2.6 to −0.3)**

*VLow-Coop, Very Low Cooperation; Low-Coop, Low Cooperation; High-Coop, High Cooperation; VHigh-Coop, Very High Cooperation. The bold on the tables are referred to the values with effects size on the different variables and the ones consider for discussion.*

## Discussion

The aim of this study was to evaluate the effects of the unbalanced number of players (4v2, 4v3, 4v4, 4v5, and 4v6) in ball possession SSGs, on cooperation behaviors between players, and in relation to opponents, on U23 soccer players’ action capabilities performance. In general, players covered more distance at higher intensity when playing in inferiority, which contributed to perceiving these conditions as more intense. In contrast, in superiority there was an increase in the distance covered while walking. In addition, playing in inferiority, and specifically during the 4v5, a decrease in the number of passes with the dominant limb was found. Coaches should then be aware of the impact of a numerical unbalance when using ball possession SSGs in their training sessions; the tendency is to increase the intensity of the exercise (external workload and RPE) as the unbalanced difference becomes higher while having similar tactical individual actions.

From an opposition-based perspective, as expected, players tend to cover more distance while walking and less while running in balanced formats when facing High-Sup and Sup. This is in line with Sampaio et al. (2014) who point out that players tend to cover more distances at lower intensities and less at higher intensities when in numerical superiority. At this level of opposition (4v2 and 4v3), players have less pressing from opponents and more time for decision making, facilitating the balance at the defensive phase and a better creation of opportunities to stay at the offensive phase by retaining ball possession (Vilar et al., 2013).

On the other hand, players in Inf and High-Inf covered more distances while sprinting, achieved a higher max speed, and walked less. Accordingly, it is likely that when in inferiority situations (4v5 and 4v6), the opposing team have a greater chance to maintain ball possession as a result of the additional(s) free player(s), increasing the distance covered at higher intensities as a result of an attempt of the team in inferiority to recover the ball and cover possible passing lines.

It was also expected that individuals at this stage of players’ development should be able to have a better tactical knowledge and understanding of the different affordances according to SSGs manipulations (Praça et al., 2015). Clearly, in High-Sup and Sup (4v2 and 4v3), players have more space and can spread around the playing area to easily keep ball possession; in Inf and High-Inf (4v5 and 4v6), players need to cover more distances at higher intensities to recover possession, due to a higher playing area density (playing area divided by the number of players).

Small effects on tactical individual actions for High-Sup, Sup, and High-Inf (4v2, 4v3, and 4v6), and moderate effects for Inf (4v5) was also observed. Contrary to what was observed (a slightly higher number of passes in 4v3 than 4v2), Vilar et al. (2014b) reported a higher number of passing and goal opportunities on 5v3 formats, but not on 5v4. However, their study included goals, which leaded to a game direction, and consequently an awareness to keep scoring goals, the reverse of a ball possession task. During a possession-based SSG, the team with numerical inferiority, within a 4v2, format may be looking to delay, working as a unit and waiting for the right moment to press as a defensive strategy due to the lower number of players. This defensive strategy provides time and space for the team with numerical superiority to retain the ball and to pass only when the opponents decrease the distance to the player in possession.

This finding is also supported by the external load results: fewer number of passes and larger distance covered while walking, due to the discussed lower need of moving by the team with numerical superiority. In contrast, during the Sup situation (4v3) there is at least one available passing line as a result of the additional player, which may afford the team with the numerical advantage to pass the ball more often to retain possession. Studies from Vilar et al. (2014a) also pointed out that, even without intercepting the ball or having a direct involvement at the play, defenders’ position can pressure attackers to make a pass earlier or to shoot at the goal. So, players’ perception and action capabilities during this unbalanced format, with a higher number, can facilitate the learning process to adapt positioning and to create passing lines, as well as a better individual game role understanding to act (Gonçalves et al., 2016), which during the context of practice is similar to the performance context, and allows players to understand the available sources of information (Davids et al., 2005; Woods et al., 2020).

Contrary to Torrents et al. (2016), results showed higher distances covered while running, sprinting, max speed and higher scores of RPE, and less distance walking, with a higher number of total players involved in the task (4v5 and 4v6). On these opposition-based formats, by decreasing playing area density, the available free space is reduced and consequently players need to perform more intensive actions to create passing lines and to control the space of play. Therefore, formats of 4v5 and 4v6 can be used to increase the physical soccer components, while at the same time allowing players to develop specific game situations under specific spatial-temporal demands.

From a cooperation-based perspective, formats of VLow-Coop and VHigh-Coop (4v2+0 and 4v2+4) allow for covering more distances while walking for the team with numerical superiority, and more distances while running and sprinting for the team with numerical inferiority. It is in line with Hill-Haas et al. (2010) who pointed out that unbalanced formats increase the number of sprints, total distance covered, and acute physiological responses on tasks for teams with numerical inferiority. Sampaio et al. (2014) also revealed that formats with numerical superiority make players cover more distances while walking with lower intensities. This can lead to an important stimulus to develop energy systems in soccer: coaches can simultaneously develop aerobic and anaerobic energy systems in the same SSGs by varying the opposition number (two extra players) and cooperation level (0: 4v2 or 4: 4v6) between players.

At the same time, a higher max speed was achieved in VHig-Coop by the numerically inferior team. Following our results, Travassos et al. (2014b) revealed that a team’s defending, in an outnumbered scene, tends to decrease the interpersonal distances between players and to the center of the team, while the attackers tend to disperse; as the difference of the unbalanced number of players gets higher, more restrictions would appear for the defensive team, who moves to their defensive goal with less action capabilities solutions. Therefore, teams with a numerical superiority may keep ball possession, spread on the playing area, and increase distances between players, taking advantage of balance tasks, and creating passing lines. Consequently, this action causes the opponent team, with a numerical inferiority, to cover more distances at higher intensities and for longer, putting more effort into the task to recover the ball.

In terms of tactical individual actions, it was only found to be trivial and had small effects for all analyzed cooperation-based formats. However, a tendency to have a higher frequency of tactical individual actions was observed for teams with a numerical superiority, maybe due to the higher number of possibilities for passing actions, created by the higher number of players in the playing area. Based on previous research, it is well known that increasing the number of players will increase the positional behavior of players on the playing area due to higher control of the space of play (Gonçalves et al., 2017). The increase in the tactical individual actions is suggestive of the adaptability of players to the space of play and the control of the passing action according to the task constraints (Davids et al., 2005).

In the same line of reasoning, teams with a numerical superiority would prefer to promote a less exploratory behavior and game variability; this easier game situation can promote more regular and less variable plays, while a more difficult scenario (numerical inferiority), may stimulate the players to explore more and new technical-tactical solutions to ensure the control of the space of play (Torrents et al., 2016). Hence, in order to promote tactical individual learning, it is necessary to develop low complexity tasks (3v1 or 5v2) (Praxedes et al., 2018), promoting the practice under easier game conditions for the offensive phase. In any of these situations, while players actively participate in learning, they are able to focus on exploring potential important sources of information rather than independently meeting the task demands prescribed by the coach. This active involvement in practice provides a basis on which coordinative structures can be set up in the early stages of learning so that later in practice they can be strengthened and optimized for skillful performance (Davids et al., 2005; Woods et al., 2020).

As expected, players with the numerical inferiority perceived the exercise as more intense, showing that the different levels of cooperation influences the intensity of the task (Kalapotharakos et al., 2011). It can be also observed that players with a higher external workload were the ones who perceived the task as more intense, contrary to the results of Hill-Haas et al. (2010), who stated that, although teams with a numerical inferiority perceived the exercise as more intense, there was no significant differences on the external workload.

Overall, the study results revealed that different physical and individual tactical actions emerge as result of the manipulation of the available number of players (opponents and teammates) on the task. From this perspective, coaches may promote possession SSGs with a difference of two players (High Sup/VLow-Cop, 4v2; and Low Inf/VHigh-Coop, 4v6) to decrease the physical demands for the team with the numerical advantage, while in turn increasing the physical stimulus for the outnumbered team. Accordingly, this type of condition may be used by coaches in the first training session of the week to increase the stimulus of substitute and non-team sheet players, while promoting a low physical and cognitive demanding task (active recovery) for line-up players. In addition, for training tasks aiming to develop players’ individual tactical actions capabilities, coaches may promote smaller unbalanced formats by using possession games with a difference of just one player between teams (Sup/Low-Cop, 4v3; and Inf/High-Coop, 4v5).

Furthermore, coaches may adjust the task complexity according to players’ capabilities by adjusting the number of players involved, while maintaining unbalanced situations (e.g., for players with lower tactical knowledge opt for 4v2+0 and 4v2+1, while for players with higher tactical knowledge use 4v2+3 and 4v2+4). Thus, ball possession SSGs with an unbalanced number of players, can be used in the coaches’ weekly planning as they contain specific offensive and defensive phases that spontaneously occur in the game, which is more realistic, physically and mentally demanding (Bekris et al., 2012), and a task which represents a dynamic system composed by many interactive constraints as found in an official match (e.g., teammates, opponents, playing area, ball) (Davids et al., 2005).

Whilst this study shows important and practical findings, some limitations must be acknowledged. It was also reported that different team sports outcomes and aspects of play depend on several factors, and therefore it is not coherent to generalize a specific drill to different competitive levels or specific game positions (Lupo and Tessitore, 2016). This investigation considered only possession-based SSGs, so different results would emerge during SSG with goals. Likewise, GPS technology has been used allowing a fast, valid, and reliable tool to obtain data of physical load during training; however, sometimes the results are inaccurate when there are rapid variations in movement over a short period of time (Malone et al., 2017). Future research should be conducted to analyze SSGs with an unbalanced number of players in different playing area dimensions, to understand the effects of the manipulation of space on soccer players’ performance. Another noteworthy investigation would be the exploration of the age-category effects while playing SSGs in unbalanced numbers and the outcomes of the learning process for young players.

## Conclusion

In summary, from an opposition-based perspective, High-Sup (4v2) and Sup (4v3) conditions allow players to walk more, while High-Inf (4v6) causes players to sprint longer distances. Player behavior adapts to the number of players involved during SSGs: when players are in numerical superiority, they can use the available space and the team’s playing area dispersion to facilitate ball possession; on the other hand, when players are in numerical inferiority, they need to increase intensity levels and perform in coordination with their teammates to recover ball possession. From a cooperation-based perspective, players tend to intensify their external workloads when the level of cooperation is null [VLow-Coop (4v2+0)] and control game pace on a VHigh-Coop condition (4v2+4). Therefore, when in lower numbers, players need to auto-organize themselves to keep a compact unit and to find the right moment to recover possession; with an increase in teammate numbers, more players are available to perform balance and cover tasks, allowing for better game control.

The findings of this study allow coaches to understand performance and behavior effects when manipulating numbers in ball possession SSGs to generate unbalanced scenarios. SSGs with numerical inferiority were more physically demanding and players perceived the exercise as more intense; from an opposition-based perspective, the higher the numbers involved in a task, the higher the RPE; from a cooperation-based perspective, the higher the level of cooperation, the smaller the RPE. Unbalanced numbers in SSGs then generate different opposition and cooperation responses to players’ performance and action capabilities, especially with a difference of two players per format.

## Data Availability Statement

The raw data supporting the conclusions of this article will be made available by the authors, without undue reservation.

## Ethics Statement

The studies involving human participants were reviewed and approved by the Ethics Committee of University of Beira Interior. The patients/participants provided their written informed consent to participate in this study.

## Author Contributions

NN and BT contributed equally to all, including data collection and data analysis. BG contributed to design, data analysis, discussion, and tables and figures. DC contributed to introduction, data analysis, discussion, and figures. All authors contributed to the article and approved the submitted version.

## Conflict of Interest

The authors declare that the research was conducted in the absence of any commercial or financial relationships that could be construed as a potential conflict of interest. The reviewer YC declared a past collaboration with one of the authors BG to the handling editor.
